# Psychometric properties of the Attitudes Scale facing Alcohol and Alcoholism in nursing students**^1^**

**DOI:** 10.1590/1518-8345.0774.2823

**Published:** 2016-12-19

**Authors:** Divane de Vargas, Fernanda Mota Rocha

**Affiliations:** 2 PhD, Associate Professor, Escola de Enfermagem, Universidade de São Paulo, São Paulo, São Paulo, Brazil; 3 MSc, RN, Hospital Alemão Osvaldo Cruz, São Paulo, SP, Brazil.

**Keywords:** Scales, Students, Nursing, Validation Studies, Psychometrics

## Abstract

**Objective:**

to verify the psychometric properties of the Attitudes Scale facing Alcohol and Alcoholism (EAFAA) and people with disorders related to the use of alcohol in nursing students.

**Method:**

a convenience sample (n=420) completed the EAFAA, the data were submitted to Exploratory Factor Analysis (EFA) and Confirmatory Factor Analysis (CFA).

**Results:**

the EFA resulted in an instrument composed of 48 items divided into four factors. The CFA has established the validity of the factorial structure. The internal consistency of the scale was considered adequate (α=0.85) presenting a sensitivity of 70% and specificity of 75%.

**Conclusion:**

the EAFAA constitutes a reliable instrument to identify the attitudes of nursing students towards alcohol, alcoholism and persons with disorders related to alcohol use.

## Introduction

Classical theories of social psychology^1^ argue that attitudes are formed and developed from social relations, not limited only to the visible behavior but also to everything that can be logically inferred from the external behavior. Also according to these theories^1^ an attitude is an enduring organization of beliefs and general cognitions, endowed with emotional charge, for or against a defined social object, which predisposes to an action consistent with the cognitions and emotions related to this object, in the case of multidimensional constructs involving affective, cognitive and behavioral components.

Given the diversity of definitions of the term attitudes, at least three common elements can be found in most of them, namely: attitudes are conscious or unconscious mental state, a value, a belief or sentiment facing a particular object, a predisposition to a certain behavior and or action^2^. 

Regarding the attitudes of nurses towards alcohol and associated issues, according to scholars of the subject^3^, these are influenced by the values and moral perceptions internalized during childhood and also in daily life. These values lead to conceiving persons with disorders related to alcohol use as without character, and guilty of their health problems, it also evokes fear in childhood, which is turned into rejection and avoidance in adulthood. Thus, the concepts and predispositions acquired in relation to those people in the social, emotional and intellectual development of the individual bring up other values, in the case of nurses, manifested through negative attitudes to people with disorders related to alcohol use^3^. 

Studies investigating the issue of training nursing students in the field of psychoactive substances have shown that most of them are often faced with people with disorders associated with the use of alcohol and other drugs, in various specialties during their training^4^. Students can reproduce the attitudes of practitioners facing this clientele, which have been described as negative and permeated by the moral connotations of the problem^3^. This phenomenon is not clear, given the scarcity of national scientific literature seeking to identify the perceptions and attitudes of nursing students towards the patient with disorders related to alcohol use^5^. 

North American studies, mainly in the last three decades of the last century, provide evidence that interventions made with nursing students through training sessions are effective in increasing knowledge; however, changes in attitude occur less frequently, and when they occur, they linger for short periods of time^6^ ^)^ ; there is also evidence that after appropriate training sessions nursing students feel safer and better prepared to intervene with alcoholic patients with more positive attitudes^6^. A more recent study^7^ found that after a theoretical training on the approach towards patients with disorders related to alcohol use, improvement was observed in attitudes relating the perception of legitimacy and capacity to work with alcohol-related problems patients among students, however, they manifested less motivation to work with this clientele at the end of training.

In Latin America and specifically in Brazil, studies on the attitudes of nursing students regarding alcohol issues suggest positive that they have positive attitudes ^4^ ^-^ ^5^, even though they prefer not to deal with this kind of patient^6^. These difficulties have been attributed by the researchers of this phenomenon^4^ ^-^ ^6^ to the sparse attention given to the teaching of this subject in the curricula of Brazilian nursing schools.

It must be emphasized, however, that despite their significant contribution to the knowledge of this phenomenon in Brazil, national studies have obtained their results using instruments mostly not validated for use among nursing students, a fact that can contribute to biases in their findings.

Given the scarcity of Brazilian studies assessing the attitudes of nursing students to issues related to alcohol^6^, to the need to map the situation in geographic areas not yet explored, and also the importance of identifying the attitudes of this population to propose strategies to characterize the attitudes of future nurses. The availability of a robust and reliable instrument can be considered essential to systematically study the attitudes of nursing students when facing issues related to alcohol and alcoholism.

The scale of attitudes towards alcohol, alcoholism and people with disorders related to the use of alcohol (EAFAA), was developed and tested psychometrically to measure this construct among nurses and health practitioners^3^ ^,^ ^8^, however, there are no studies of its psychometric properties among nursing students. 

The EAFAA consists of 50 items divided into four (4) factors: Factor 1: The work and interpersonal relationships with patients with disorders related to alcohol use; Factor 2: The person with disorders related to alcohol use; Factor 3: The alcoholism (Etiology); Factor 4 - Alcoholic beverages and their use. This Likert scale has five points (1 being strongly disagree and 5 fully agree) showing reliability indices close or equal to 0.90^3^ ^,^ ^8^. 

The positively oriented EAFAA items are usually ordered in a way to avoid the acquiescence in the style of responses, i.e., the tendency to constantly endorse "agree" or "disagree" in response to the items. The estimated time to complete the instrument is 15 minutes^8^ .

Given that the estimates of the validity of instruments and its reliability depend mainly on the nature of the samples, and the fact that whenever an instrument is used in a new context or in different groups, it is necessary to re-establish its psychometric properties^9^. It also needs to be considered that the psychometric studies published on EAFAA result of its application in health practitioners^3^ ^,^ ^8^. This study aimed to study the psychometric qualities of EAFAA among nursing students.

## Objective

Verify the psychometric properties of the Attitude Scale toward Alcohol, Alcoholism and people with disorders related to the use of alcohol, in nursing students.

## Method

420 nursing students of the fourth and third year were recruited from three nursing schools (two private, one public) in São Paulo during the period of January 2011 to December 2012. Participants were randomly divided into two samples, one of them composed by 75% of participants (n=298) and the second consisting of the remaining 25% (n=122). In order to preserve the anonymity of the institutions, the schools are named as School A School B and School C. The collection instruments were constituted from the EAFAA composed of 50 items^8^ and a sociodemographic questionnaire with information on age, gender and year (third or fourth) attended at the time of collection. 

For data collection, the questionnaires were distributed in the classroom in envelopes to be deposited in an urn in the back. The participants who agreed to participate in the research deposited the answered instruments without identification. The Research Ethics Committee of the institution of the main researchers approved the proposal prior to its beginning. Of the respondents, the majority, 61%, were from School A. Table 1 shows the demographic data of the participants according to the Exploratory Factor Analysis (EFA) and Confirmatory Factor Analysis (CFA).

**Table 1:** Factorial matrix of the EAFA. São Paulo, SP, Brazil, 2015

		F1	F2	F3	F4
01	I'm afraid to address the alcohol problem with my patients.	.56			
05	I fear the aggression of patients with disorders related to alcohol use.	.44			
09	I feel frustrated when working with patients with disorders related to alcohol use.	.48			
13	Of all my patients, patients with disorders related to alcohol are the ones that make me work the most.	.48			
17	I have to take care of patients with disorders related to alcohol use, even if they believe that they do not need health care.	.57			
21	Even when not intoxicated, patients with disorders related to alcohol use are disrespectful to the team members.	.55			
25	I feel angry when working with patients with disorders related to alcohol use.	.55			
29	Patients with disorders related to alcohol use will never accept health professionals talking about their drinking problems.	.51			
37	Addressing the alcohol problem in patients with disorders related to alcohol use means less time for other patients.	.43			
41	I prefer to work with patients with disorders related to alcohol use instead of working with other patients.	.46			
42	The person with disorders related to alcohol use is a difficult person to relate to.	.70			
44	I find it hard to establish a therapeutic relationship with patients with disorders related to alcohol use.	.72			
46	We must be careful not to be assaulted when working with patients with disorders related to alcohol use.	.52			
48	When patients with disorders related to the use of alcohol do not accept having problems related to alcohol use, the best decision is to give up helping them.	.46			
49	When working with patients with disorders related to alcohol use, I do not know how to handle the situation.	.49			
50	Caring for patients with disorders related to the use of alcohol is not rewarding for me.	.55			
	Number of items 16 -Variance explained 23,1%				
02	People with disorders related to alcohol use have no common sense.		.49		
06	People with disorders related to alcohol use are rude.		.62		
10	People with disorders related to alcohol use are irresponsible.		.61		
14	Patients with disorders related to alcohol use are more likely to become violent against me.		.63		
18	I believe that people who develop alcoholism are weak.		.54		
22	I notice that patients with disorders related to alcohol use do not want to help themselves.		.56		
26	I do not trust the information reported by patients with disorders related to alcohol.		.62		
30	I believe that persons with disorders related to alcohol abuse are guilty for their health problems.		.50		
34	Persons with alcohol-related disorders always end up coming back to the health service with the same problem.		.53		
38	Of all my patients, patients with alcohol-related disorders are the most difficult to handle.		.46		
45	Patients with alcohol-related disorders are patients who cooperate with their treatment.		.56		
47	People with disorders related to alcohol use do not take their treatment seriously.		.61		
	Number of items 12 -Variance explained 8.2%				
03	I believe that going through family problems leads to alcoholism.			.65	
07	Shy or inhibited people are more likely to develop alcoholism.			.47	
11	I believe that depression leads to alcoholism.			.58	
15	Willpower is missing in people with alcohol-related disorders.			.45	
19	Social issues lead the individual to drink.			.55	
23	Hereditary predispositions lead to alcoholism.			.44	
31	People who develop alcoholism have low self-esteem.			.50	
35	People with alcohol-related disorders are psychologically shaken.			.41	
39	People drink to feel more sociable.			.45	
43	Persons with alcohol-related disorders drink because they cannot face their reality.			.62	
	Number of items 10 -Variance explained 5.8%				
04	I think people have the right to drink if they want.				.59
08	Alcoholic drinks are nice and they it provides a wellbeing sensation to the user.				.72
12	The use of alcoholic beverages is normal.				.75
16	Drinking in any amount will make the individual dependent.				.48
20	Drinking in moderation is not harmful.				.59
24	I am against the use of alcohol at any time.				.42
28	I am in favor of moderate drinking.				.71
32	Small doses of alcohol are capable of causing dependence.				.58
36	The use of alcohol in small quantities is beneficial.				.59
40	People can drink provided they can control themselves.				.66
	Number of items 10 - Variance explained 4%				

Given that it was an instrument already validated in populations of nurses and other health professionals, the data were submitted to a Confirmatory Factor Analysis (CFA) in a first step, obtaining a poor adjustment of the model. In view of this result, the data were submitted to an Exploratory Factor Analysis (EFA) after verifying whether the data met the criteria of normality and sphericity through the Kaiser-Meyer-Olkin test and Bartlett's test of sphericity. The data of sample 1 (n=298) underwent Exploratory Factor Analysis (EFA) with main axis extraction and Oblimin rotation .

The criteria adopted for the composition of the factors and for keeping items in the model were the same used in validating the original version of EAFAA^8^. This standard has also been followed to determine the reliability of the scale, using the Cronbach's alpha for the instrument in its entirety and for each of the extracted factors. The Pearson correlation coefficient was used to verify the correlation between the variables of EAFAA, and between the scores obtained by the sample in the instrument before and after the changes brought about by the AFE.

The cutoff of the EAFAA nursing students version, was identified from the analysis of the ROC technique (receiver operating characteristic), and defined as the one that maximized the Youden index^10^.

The best model identified by the AFE was the result of the extraction of 4 factors (after exclusion of items with no significant factor load and with double factor load) this model was subsequently selected for the Confirmatory Factor Analysis using the second sample (n=122). The CFA was conducted using the Analysis of Moment Structures method (AMOS) version 22, and the following indexes adjustments were examined: Tucker-Lewis index (TLI), Comparative Fit Index (CFI) and the Root Mean Square Error of Approximation (RMSEA). We also assessed the parsimony of the model through the parsimony comparative fit index (PCFI) and the parsimony normed fit index (PNFI).

## Results

The criteria of normality and sphericity checked by the Kaiser-Meyer-Olkin and Bartlett's sphericity tests were attended presenting respective values of (0.85) and significance of (<.0001). The model consists of 50 items distributed in 4 factors and it was the best solution for this version of the scale. The model was submitted for consideration, with the exclusion of 2 items that made up the initial version of the instrument^8^ (F2_33 -_I consider the patient with disorders related to alcohol use as a lost cause & F3_27 -_ Dissatisfied people abuse of alcohol) because they have a factor load below the determined cut-off point to stay in the instrument (0.40), and because they have shown significant factor load on more than one factor at the end of the rotation.

Excluding these two items from the EAFA for nursing students did not cause damage to the reliability coefficients of the respective factors and the EAFAA. The observed result was consistent with the results of the factor analysis of the original scale^8^, keeping 48 items distributed in 4 factors (Table 1). 

The reliability coefficients measured by Cronbach's alpha for the scale in full (α=0.85) and also for each of the 4 individual factors were considered satisfactory in both cases (Table 2), with a significant correlation between the factors and between these and the full version of the scale (Tabela 2). As expected, a significant correlation was found (r=0.90, p<000.1) between the scores obtained by the sample in EAFAA^8^ and in the version modified by the EFA, supporting the validity criteria of the EAFAA for nursing students.

**Table 2:**
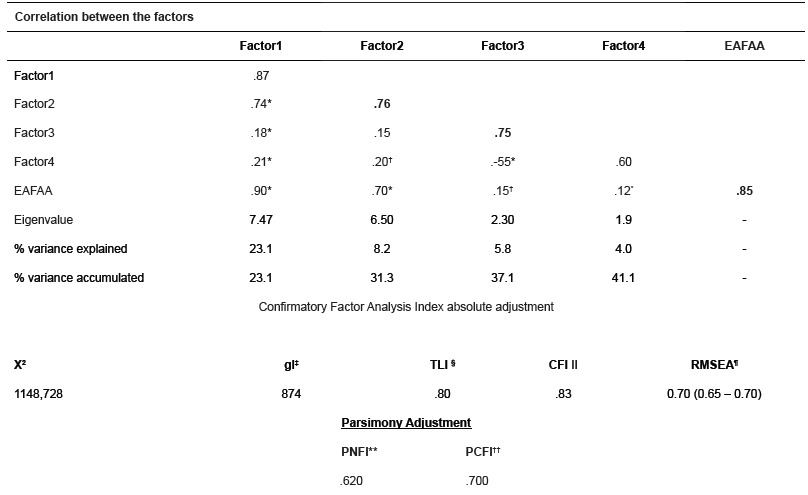
Description of the correlations between the factors that make up the EAFAA and psychometric characteristics resulting from the Exploratory Factor Analysis and Confirmatory Factor Analysis. São Paulo, SP, Brazil, 2015.

*Significant correlation to the level 0.01; † significant correlation to the level 0.05;‡ degrees of freedom; §. Tucker- Levis Index; II Comparative Fit Index; ¶ Root Mean Square Error of Approximation; **Parsimony Normed Fit Index; †† Parsimony Comparative Fit Index.

Elements in bold in the main diagonal of the correlation matrix between the factors correspond to the reliability factor.

The analysis for the selection of the cut-off point of the EAFAA adopted from the ROC curves technique pointed out as optimal for nursing students the 3.29 score presenting a sensitivity of 70% and specificity of 75%.

**Figure 1:**
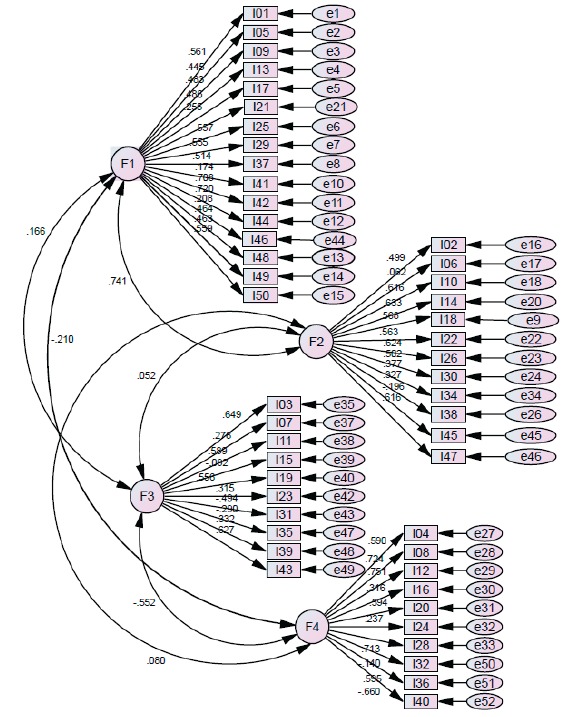
Resulting path diagram of the Confirmatory Factor Analysis. São Paulo, SP, Brazil, 2015

The model with four factors resulting from the EFA, was assessed in the second sample of participants (n = 122) using the CFA for this purpose. The results of this analysis showed reasonable fit of the model as suggested by the indexes (Table 2). The reduced model is shown in Figure 1. All four factors presented inter-correlation among them (Table 2).

## Discussion

The results of this study provide evidence to support the validity of the EAFAA for use among nursing students, the factorial structure of the resulting scale of the AFE is consistent with that obtained in the original version^8^. Although composed of 48 items, two less than in the previous version^8^, the EAFAA maintained unchanged the composition of the four factors; Factor 1: Work and interpersonal relationships with patients with disorders related to alcohol use; Factor 2: The person with disorders related to alcohol use; Factor 3: O alcoholism-(Etiology); Factor 4 - Alcohol and its use, observing a significant correlation coefficient with the scores observed in the health practitioners' version^8^. 

The internal consistency of the EAFAA for nursing students, as established by Cronbach's Alpha, showed adequate levels of reliability for the full version as well as for each one of its four factors^11^. The factors 1, 2 and 3 showed excellent, good and adequate indexes respectively of reliability^11^, the factor 4 showed acceptable reliability index (α=0.60)^11^, consistent with previous studies that evaluated the psychometric properties of the EAFAA in different populations^3^ ^,^ ^8^. It was also noted a low correlation of this fact with the EAFAA and the other factors of the scale, a result also observed in previous studies^8^

Among the reasons for this result, we can highlight the low variability in factor 4 in this version of the EAFAA (4%), providing additional evidence to the idea that this factor might be measuring another construct^8^. This can only be established more positively through studies aimed to test this hypothesis^8^.

The results suggest that the EAFAA has a satisfactory structural validity, since it presented 40% of the total variance of the data, concentrating over 20% of it in the first factor^12^, matching consistently with what was observed in the original version^8^ i.e. the one-dimensional characteristic of the scale^12^ ^-^ ^13^. 

The analysis of sensitivity and specificity of the EAFA showed that it has 70% (p < .000) of probability of identifying individuals who score above 3.29 on the scale and that actually have a positive attitude towards alcohol, alcoholism and people with disorders related to alcohol use. This cutoff point is slightly higher than that observed in the version for use among health professionals (3.15)^8^.

The hypothesis that the four correlated factors retained by the AFE are reliable and valid was confirmed by the AFC by the setting rates and parsimony, with values close to the cutoff point suggested by the literature which are, in the case of absolute fit indices: Comparative Fit Index (CFI) >0.85^14^; Tucker- Levis Index (TLI) >0.90^15^ and the Root Mean Square Error of Approximation (RMSEA) < 0.08. Although the absolute fit indices have been performing below the cutoff point established by some authors, we need to consider that there is no ideal index representing a definitive criterion for testing a structural model^16^. Therefore it is recommended that the results are assessed in a selected set of fit adequacy ratios, even if there is no consensus among all^17^. Based on these assumptions, it can be said that evaluating together the results of CFA, they reject the null model, suggesting satisfactory fit of the model, since it is a parsimonious model presenting PCFI and PNFI ≥ 0.60^16^, and RMSEA <0.08.

All factors presented correlation among each other, the positive correlations observed between factors 1 and 2 suggest that the scores of these two factors are positively correlated which may imply that the scores on the factor that measures attitudes towards work and interpersonal relationships with people with disorders related to alcohol use is correlated with the score obtained related to people with disorders related to alcohol use. This means that subjects who get high scores on factor 1 tend to also score high in factor 2, being the opposite also true^8^. 

Consistent with previous studies^3^ ^,^ ^8^ the EAFAA for nursing students is also positively oriented, which means that with the exception of items F1_17_, F1_41_, F3_03_, F3_07_, F3_11_, F3_15_, F3_19_, F3_23_, F3_27_, F3_31_, F3_39_, F4_04_, F4_08_, F4_12_, F4_16_, F4_20_, F4_28_, F4_36_, F1_17_, F3_03_ , F3_11_, F3_19_, F3_23_, F3_35_, F3_39_, F4_04_ , F4_08_, F4_12_, F4_20_, F4_28_, F4_36_ the answers to all others must be calculated with inverted values, thus the scores should be calculated as follows: (1=5), ( 2=4), ( 3=3), (4=2),(5=1). 

This study provides important contributions to nursing. Within the scope of research, it provides the scientific community with a psychometrically tested instrument for use among nursing students, filling a gap in this area. The availability of this version of EAFAA can also contribute to the increase of research on this subject in the country, and it can also be the subject of further research aiming to validate it in other cultural contexts. It may facilitate to identify common and divergent aspects in the attitudes of nursing students in different settings and cultures.

Among the implications for the nursing practice, the EAFAA can be used to assist teachers in identifying the attitudes of future professionals, thus enabling them to direct their actions in order to change them, in cases where the results of its application reveal negative attitudes. The EAFAA can even be used for the identification of the effects of proposed strategies to change the attitudes of students, comparing different groups and interventions.

Having certainty that the EAFAA has adequate levels of reliability and validity, it is suggested that the scale presents robust indexes of validity for use in nursing students' samples and their results may provide subsidies for research and the practice of nursing. This study should be interpreted within the framework of several limitations, namely, the specific time when data was collected and the convenience samples that even coming from institutions with different characteristics, their origin is the same region of the country. For those reasons it is advisable to enlarge the research using EAFAA through the inclusion of the regional differences in the country.

Moreover, the fact that the sample was predominantly composed of females may have influenced the external validity of the results and future studies should include more male students. Additional psychometric studies obtained with other samples are desirable in order to test the construct validity demonstrated here.

## Conclusion 

The psychometric properties of EAFAA suggests that it is in a reliable instrument to identify the attitudes of nursing students towards alcohol, alcoholism and the person with disorders related to alcohol use. The results are the basis for the advancement of science by offering an instrument with reliable psychometric properties to measure the attitudes of this population towards the theme alcohol and alcoholism with accuracy. It is suggested that its psychometric qualities are tested in different populations and in other contexts and situations.
